# Larval Rearing and Nutrition of the Polyphagous Tephritid Pest *Anastrepha ludens* on Artificial Diets with Calcium Alginate, Agar, or Carrageenan as Gelling Agents at Various Concentrations and across Extreme Larval Density Conditions

**DOI:** 10.3390/insects14120952

**Published:** 2023-12-15

**Authors:** Carlos Pascacio-Villafán, Luis A. Caravantes-Villatoro, Ixchel Osorio-Paz, Larissa Guillén, Hugo S. García, Erick Enciso-Ortiz, Alma Altúzar-Molina, Roxana Barran-Prior, Martín Aluja

**Affiliations:** 1 Red de Manejo Biorracional de Plagas y Vectores, Clúster Científico y Tecnológico BioMimic®, Instituto de Ecología, A.C., Xalapa 91073, Veracruz, Mexico; luis.caravantes@inecol.mx (L.A.C.-V.); ixchel.osorio@inecol.mx (I.O.-P.); larissa.guillen@inecol.mx (L.G.); erick.enciso@inecol.mx (E.E.-O.); alma.altuzar@inecol.mx (A.A.-M.); roxana.barran@posgrado.ecologia.edu.mx (R.B.-P.); martin.aluja@inecol.mx (M.A.); 2 Unidad de Investigación y Desarrollo de Alimentos, Tecnológico Nacional de México, Instituto Tecnológico de Veracruz, Veracruz 91897, Veracruz, Mexico; hugo.gg@veracruz.tecnm.mx

**Keywords:** *Anastrepha ludens*, sterile insect technique, mass rearing, artificial diet, gelling agent, tephritid pest, larval density, insect nutrition

## Abstract

**Simple Summary:**

Artificial diets are one of the most critical components in insect mass-rearing facilities worldwide. Research on artificial diets to rear pestiferous tephritid flies dates back to the 1950s but is still a topic of great relevance, as fruit flies represent one of the ten most important pests of fruit in temperate and tropical regions of the world. In the past 15 years, much research has been performed to develop liquid- or gel-based diets more closely resembling the rearing environment a wild fruit fly larva experiences inside a fruit in nature. To identify new gelling agents that could perform better than already established ones such as agar and carrageenan, we assessed the performance of calcium alginate, a gelling agent widely used in the food industry, that had not been tested in the case of fruit fly mass rearing. We also examined larval density in gel diets as a critical factor that affects insect-rearing systems. Our results show that agar and carrageenan diets outperformed calcium alginate diets broadly and that the effects of larval density depended on the gel type used. We believe that the applications of calcium alginate in tephritid rearing should be analyzed in more depth in future studies.

**Abstract:**

Research on larval rearing and nutrition of tephritid flies on artificial diets is key for the sterile insect technique. Here, we examined the effects of the type of gel (calcium alginate, agar, or carrageenan), at varying percentages in artificial diets for the polyphagous pest *Anastrepha ludens*, on the physicochemical and nutritional traits of the diets, and the effects of the type of gel, the gel content and the larval density (larvae/g of diet) used in production, quality parameters for mass-reared tephritids, diet removal (an indirect estimation of diet consumption), and nutritional traits of flies. Regardless of the gel content, calcium alginate diets were firmer and more resistant to penetration than the agar and carrageenan diets. The larval recovery, pupation, pupal weight, and flight ability of *A. ludens* were lower in calcium alginate diets than in agar and carrageenan diets. Diet removal was higher in calcium alginate diets; however, low levels of ammonium and high levels of uric acid in excretions from larvae on these diets suggest an alteration in protein metabolism. The firmness and penetration resistance characteristics of calcium alginate diets may have limited movement and feeding of larvae, but this could be overcome by the collective feeding of large groups of larvae. Our findings provide insights into the mechanism governing gel-diet rearing systems for *A. ludens*.

## 1. Introduction

One of the most important polyphagous tephritid fly pests of quarantine and economic importance from southern Texas, USA to Costa Rica is the Mexican fruit fly, *Anastrepha ludens* (Loew) (Diptera: Tephritidae) [1,2]. It infests a variety of commercial hosts including mango (*Mangifera indica* L. (Anacardiaceae)) and citrus (*Citrus* spp. (Rutaceae)) [3,4], and is a potential invader of temperate areas in the face of global warming [2,5,6]. In Mexico, *A. ludens* is expanding its geographical and host range to infest apples (*Malus × domestica* Borkh. (Rosaceae)) in high-altitude temperate areas (M. Aluja, personal observations). *Anastrepha ludens* is mass reared on artificial diets for sterile insect technique (SIT) applications in Guatemala, Mexico, and the USA [7]. The SIT is an environmentally friendly method of pest control based on the artificial rearing of millions of insects followed by sterilization and mass release of sterile male insects into the field to mate with wild females, because females that mate with sterile males leave no offspring [8,9,10]. More than 19 countries around the globe have at least one mass-rearing facility to produce sterile insects for SIT purposes [7]. The Moscafrut facility in Metapa de Domínguez, Chiapas, Mexico is the largest *A. ludens* mass-rearing facility with a production capacity of ca. 170 million pupae per week [11,12]. *Anastrepha ludens* is also reared on an artificial diet to provide larval hosts for mass rearing parasitoid wasps used in biological control programs against tephritid pests [11,13].

Artificial larval diets for the mass rearing of *A. ludens* are oligidic (i.e., their ingredients are not fully chemically defined or highly purified [12,14]), and the formulation used at the Moscafrut facility contains corncob powder as a texturizing and bulking agent [11,15]. Concerns have been raised about the use of corncob powder in the *A. ludens* diet because this ingredient can vary in quality and may be contaminated with mycotoxins that affect the growth and development of larvae, hindering the achievement of established production goals [16,17]. Gel diet formulations that use some type of gelling agent instead of corncob powder have been developed and tested for *A. ludens,* with promising results for mass-rearing approaches [18,19,20]. It was proposed that gel diets may better simulate the consistency and moisture characteristics that *A. ludens* larvae find in nature in the pulp of their host fruit [20]. Simulating the natural conditions of insects reared in artificial settings can translate into a better quality of flies for SIT purposes [21]. Gel diets also have the advantage that they can be almost entirely consumed by larvae, leaving very few diet residues compared with the traditional diet formulations using texturizing and bulking agents (such as corncob powder) [20,22].

There are several factors to consider when developing new artificial diets or when optimizing existing diet formulations for mass rearing, including insect yields (i.e., individuals produced per mass of diet), the overall health and fitness of insects, labor time, and production costs (mainly the cost of artificial diet) [11,12,23,24,25,26,27]. Careful consideration should be paid to the physical, chemical, and nutritional characteristics of the diets that could influence their palatability, consumption, digestion, and absorption of nutrients by the insect [14,15,17,28,29]. Recent studies highlight the relevance of assessing nutrient metabolism in larvae and food bioconversion as indicators of the quality and suitability of diets for mass rearing [28,29]. A factor often overlooked in optimizing insect-rearing systems is the density of individuals in rearing containers [30]. However, this factor is critical in tephritid fly mass rearing because larval density in the diet and density by diet interactions affect the development and performance of artificially reared tephritid flies [31,32,33,34]. The effects of larval density can be negative when high intraspecific competition results in poor larval nutrition [32,33], positive when groups of larvae cooperate to better exploit food resources [32,34], or vary depending on the nutritional characteristics of the medium [32].

Our goals here were to advance the state of knowledge related to the larval rearing and nutrition of tephritid flies on artificial diets, and improve our understanding of the mechanisms operating in a gel-diet rearing system for *A. ludens*. To this end, we used a design of experiments (DOE) and response surface methods (RSM) approach [20,25,35,36,37] to examine main and interaction effects of the type of gelling agent and its content in an artificial larval diet for *A. ludens* on the physical, chemical, and nutritional traits of the diets. We also examined the main and double interaction effects of the type of gelling agent, the gel content and the larval density in the diet on production, quality parameters for sterile mass-reared tephritids, diet removed by larvae (as an indirect estimation of diet consumption), and nutritional traits of flies. We used agar and carrageenan as cost-effective gelling agents in yeast-reduced artificial diets that produce similar numbers of high-quality *A. ludens* flies [20] and calcium alginate as a novel gelling agent in tephritid fruit fly diets. Alginate, agar, and carrageenan are polysaccharides; the first is produced by brown seaweeds and the other two are produced by red ones [38]. The gelling mechanism of calcium alginate involves a chemical reaction between alginate molecules and calcium ions known as the “egg box junction” that occurs without the need for high temperatures, as is the case of gelation with agar and carrageenan [39,40]. Calcium alginate gels have been used to formulate artificial diets for lepidopteran larvae [41,42,43,44] and predatory insects [45,46,47]. Based on these previous reports on the effective use of calcium alginate gels in insect diets, our working hypothesis here was that calcium alginate gels would perform as well as agar and carrageenan in an *A. ludens* larval diet, and we predicted similar yields and responses for flies reared on either calcium alginate, agar, or carrageenan diets. By incorporating the physicochemical analyses of the diets, the larval density factor, the variables of production and quality, the diet removal, and the nutritional traits of flies, our study addresses current research needs that are key for improving mass-rearing strategies for tephritid fly pests for the SIT [28,29].

## 2. Materials and Methods

### 2.1. Origin of Experimental Flies

*Anastrepha ludens* were obtained from a laboratory colony established at the “Planta Piloto de Cría de Moscas de la Fruta y Enemigos Naturales (parasitoides)”, Red de Manejo Biorracional de Plagas y Vectores (RMBPV) of the Instituto de Ecología, A.C., Xalapa, Veracruz, Mexico. Details on the maintenance and rearing of this colony are described elsewhere [48,49]. In short, *A. ludens* larvae were reared on an artificial diet [48,49] and, after pupation, pupae were placed in 30 × 30 × 60 cm Plexiglas cages. Adults emerged inside cages and were provided with food (a 3: 1 mixture of cane sugar: yeast hydrolysate MP Biomedicals, LLC, Solon, OH, USA) and water ad libitum (water was administered through a felt wick inserted into a plastic container with water and a lid). The adult density in cages was ca. 0.4 adults per cm^2^ (ca. 3700 adults per cage). Ten days after adult emergence, a 10 cm diameter oviposition device with transparent furcellaran gel (Burtonite 44C, TIC GUMS Inc., Belcamp, MD, USA) was placed on top of a cage so that females could lay eggs into it [48]. Eggs were collected from oviposition devices and washed twice with purified water. Then, by decanting, the water was replaced with a 0.2% (*w/v*) sodium benzoate solution. For the experiments, volumes of eggs (0.0127 to 0.1 mL) were measured in graduated microcentrifuge tubes and poured into black terylene cloth on top of a felt fabric moistened with a 0.2% (*w/v*) sodium benzoate solution inside an 8.5 cm diameter plastic Petri dish. Eggs were counted to reach specific density treatments as described in Section 2.4. Larval density treatments. Petri dishes with eggs were closed with a lid and incubated for four days in a dark laboratory at 29 ± 1 °C and 70 ± 5% relative humidity (RH).

### 2.2. Experimental Design

**Figure 1:**
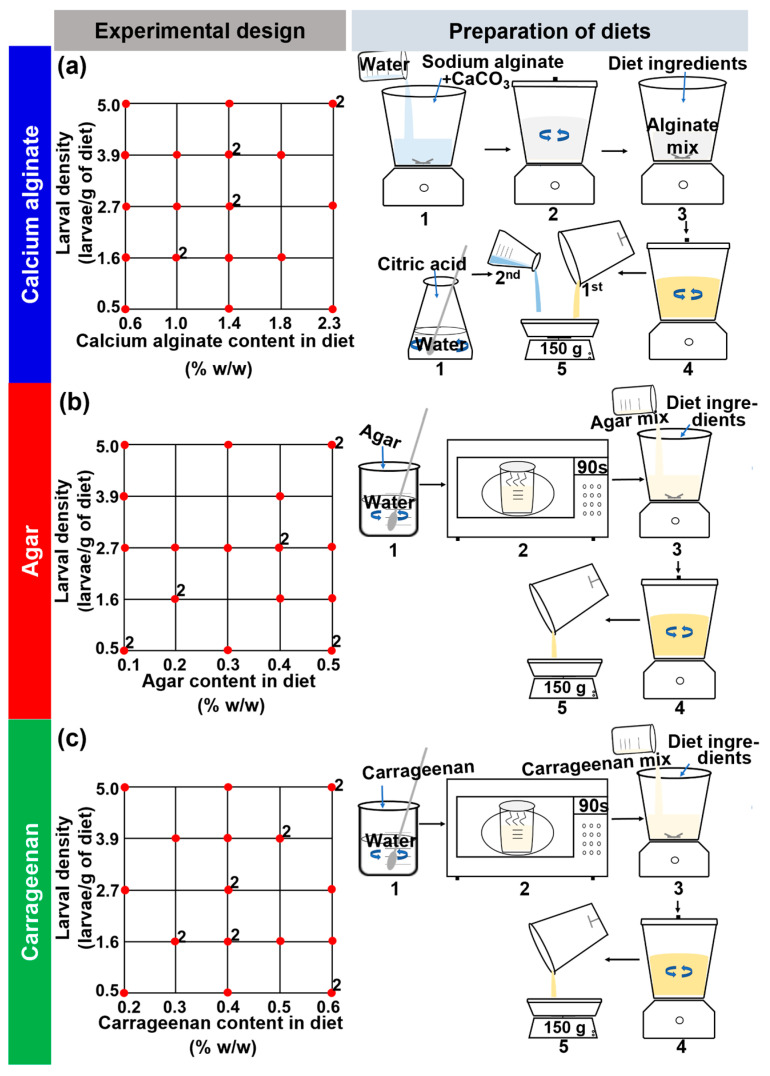
Schematic representation of the experimental design and the preparation of gel diets with (**a**) calcium alginate, (**b**) agar, and (**c**) carrageenan. The figures on the left under the label “Experimental design” depict the design space constructed for each gelling agent (calcium alginate, agar, and carrageenan) defined by the upper and lower limits of the gel content and larval density in the diets, the red points indicate the gel content × larval density combinations tested, and the number next to some points indicates the number of replications of that particular point. Note that the lower and upper limits of the gel content in the diets and the larval density are presented here in actual units (i.e., as a percentage in the case of the gel content in diets and as larvae per g of diet in the case of larval density) but we used coded units (from −1.0 to 1.0) for experimental and statistical modeling purposes, as explained in Section 2.2. Experimental Design. In the figures on the right under the label “Preparation of diets”, the numbers 1–5 indicate the diet-making steps that are described in detail in Section 2.3.1. Preparation of Diets.

We designed a response surface experiment with the Design-Expert^®^ 10 (StatEase Inc., Minneapolis, MN, USA) software [50]. The explanatory variables were categorical—type of gelling agent with three levels (calcium alginate, agar, and carrageenan)—and continuous—content of the gelling agent in the diet (% *w/w*) and larval density in the diet (larvae per g of diet) (Figure 1). The minimum and maximum percentages of calcium alginate, agar, and carrageenan used in the diets and the estimated number of larvae per g of diet inoculated into them (Figure 1) were transformed into coded units for experimental design construction and analyses. The lowest percentage of each gelling agent and the lowest larvae per g of diet were set to −1.0, whereas the highest percentage of gelling agent and larval density were set to +1.0; the average percentage for each gelling agent was centered at zero. The advantage of using coded units was that the range of each gelling agent was reduced to a common scale. Using coded units is recommended to better describe response surface designs and analyses, and the coded values can be easily converted to the actual values of a response [50]. Response variables were grouped into three broad categories: (i) physical, chemical, and nutritional traits of diets, (ii) production and quality parameters of flies, and (iii) diet removed by larvae (an indirect estimation of diet consumption) and larval and adult nutritional traits (Table 1).

The design had 11 model degrees of freedom, 38 lack-of-fit degrees of freedom, and 15 degrees of freedom for pure error; sufficient to detect linear, two-factor interaction, and quadratic effects of the explanatory variables on the response variables. Treatments (diet × larval density combinations) were randomly assigned to the experimental units (i.e., 18 cm long by 9 cm wide by 5.5 cm high rearing trays) and the experiment was performed in one block. The experiment consisted of 65 experimental runs from which 23 response variables (Table 1) were measured and modeled.

### 2.3. Diets

Diets were based on previous formulations for *A. ludens* with agar (Industrias Ragar S.A. de C.V., Coyoacán, CDMX, Mexico) or carrageenan (Industrias Ragar S.A. de C.V., Coyoacán, CDMX, Mexico) as the gelling agent and were designed to test a range of consistencies from soft to firm gels [20]. In addition to agar and carrageenan, we tested calcium alginate as a novel gelling agent in tephritid fruit fly diets. We used sodium alginate (SHQ, Suministros y Herbolarios Químicos, S.A. de C.V., Atizapán de Zaragoza, Edomex, Mexico) and calcium carbonate (CaCO_3_) (Medimart, Nueva Wal-Mart de México, S. de R.L. de C.V., Azcapotzalco, CDMX, Mexico) to form calcium alginate gels. The presentation of the calcium carbonate that we used was in tablets, and these were pulverized in a mortar with a pestle before use in experiments. All the gelling agents used were food grade. We tested 15 artificial diet formulations (five for each type of gelling agent) all with the same level of nutritional ingredients and variations in the content of the gelling agent and water; citric acid content varied only in the case of calcium alginate diets (Table 2).

Eighty percent of the calcium alginate diets consisted of varying quantities of calcium alginate (sodium alginate:CaCO_3_ in a proportion of 0.7:0.3 [55]), water, and citric acid (Table 2). The remaining 20% of the diets consisted of constant levels of yeast and cane sugar as main nutritional ingredients, corn flour as a bulking agent, and sodium benzoate and methylparaben as preservatives (Table 2). The calcium alginate percentage in the diets varied from 0.57 to 2.28% and water was reduced from 79.26 to 77.05% as the calcium alginate increased (Table 2). Citric acid was used as an acidifying agent to release calcium ions from CaCO_3_ and start the gelling process of alginate, and its content in diets ranged from 0.165 to 0.66% (Table 2). The percentage of citric acid in the diets was based on a citric acid/CaCO_3_ ratio of 0.5 mol citric acid/mol CaCO_3_ [55]. The upper and lower limits of calcium alginate percentages tested were based on preliminary tests to calibrate the use of calcium alginate in the diet and in which we obtained gels with a range of consistencies from soft to firm, similar to the touch to those known in agar and carrageenan diets.

In the case of the agar and carrageenan diets, 79.56% (*w/w*) of the diets consisted of either agar or carrageenan and water (Table 2). The remaining 20.44% of the diets consisted of constant levels of yeast and cane sugar as the main nutritional ingredients, corn flour as a bulking agent, sodium benzoate and methylparaben as preservatives, and citric acid as an acidifying agent (Table 2). The gel percentage in agar and carrageenan diets ranged from 0.12 to 0.5% and 0.2 to 0.6%, respectively, and water was reduced from 79.44 to 79.06% as the agar content increased and from 79.36 to 78.96% as the carrageenan content increased (Table 2). These ranges for the gel percentages in the diets were based on a previous study with agar and carrageenan diets [20].

#### 2.3.1. Preparation of Diets

To prepare agar and carrageenan diets (Figure 1, Table 2), the dry ingredients required to prepare 200 g of each diet were weighed individually on an analytical balance (Ohaus Explorer EX224, Parsippany, NJ, USA) and water was measured with a graduated cylinder and a micropipette. The gelling agent (i.e., agar or carrageenan) was dissolved with the total volume of water in a 250 mL beaker. The beaker containing the gelling agent dissolved in water was covered with a lid and heated in a microwave until boiling for ca. 90 s. The resulting liquid was mixed with the rest of the diet ingredients in a domestic blender (MagicBullet^®^ Express, Homeland Housewares, New York, NY, USA) for 40 s [20]. Then, 150 g of each diet were poured into 18 cm long by 9 cm wide by 5.5 cm high rearing trays for bioassays with larvae. Samples of 8 g of each diet were dispensed into 3.5 cm diameter plastic Petri dishes for firmness and penetration resistance tests, and 20 g samples were dispensed into 8.5 cm diameter plastic Petri dishes for nutrient determination and pH tests. Additional batches of 150 g of each diet (without larvae) were used to determine the fresh weight loss of the diets over time. After dispensing in rearing trays or Petri dishes, the diets were allowed to cool at room temperature for 2–22 h before they were used.

As with the agar and carrageenan diets, in the case of calcium alginate diets (Figure 1, Table 2) we used 150 g portions of diet in rearing trays for bioassays with larvae. We separated 8 g and 20 g samples for the firmness and penetration resistance test and the nutrient and pH tests, respectively, and portions of 150 g of diet for fresh weight loss determination. To prepare alginate diets, we first prepared five basic diets by mixing all diet ingredients (Table 2), except citric acid, with 90% of the total volume of water. The sodium alginate and CaCO_3_ were first blended with the water for 1 min in an industrial 3 L blender (Mixer 05M, Gustavo A Madero, CDMX, Mexico) and then the rest of the diet ingredients were added and stirred for an additional 1 min. Citric acid was dissolved with 10% of the total volume of water and maintained separately in Erlenmeyer flasks (Figure 1). The basic diets were dispensed in rearing trays or Petri dishes as indicated before and the proportional quantity of dissolved citric acid was added to reach the desired weights and initiate gelation. The diets were allowed to stand for 16–22 h at room temperature before they were used in experiments.

### 2.4. Larval Density Treatments

Larval density treatments were designed to include the range of densities used for *A. ludens* mass rearing (3.8–4.8 larvae per g of diet, [56]), to test an extremely low density not tested before (0.5 larvae per g of diet), and to experiment with a tenfold increase from the lower to the higher density [32]. As such, the estimated numbers of larvae per portion of 150 g of diet were 75, 244, 413, 581, and 750, which were equal to densities of 0.5, 1.625, 2.75, 3.875, and 5 larvae per g of diet. To reach such larval densities, *A. ludens* eggs were counted on pieces of black terylene cloth on top of moistened fabric felt inside an 8.5 cm diameter plastic Petri dish as described above in Section 2.1. Origin of Experimental Flies. Considering a mean (±SE) hatching percentage of 75.8 ± 1.97% (estimated from a sample of 10 Petri dishes with 100 eggs each), the number of eggs we counted to reach specific larval density treatments were 99, 322, 544, 767, and 989 eggs for the densities of 0.5, 1.625, 2.75, 3.875, and 5 larvae per g of diet, respectively.

### 2.5. Experimental Procedures

Dark terylene cloths containing the various larval density treatments were placed on top of the diets inside the rearing trays. Then, the trays were covered with pieces of pantyhose (Sutil^®^) and incubated in a dark laboratory at 29 ± 1 °C and 70 ± 5% relative humidity (RH) for nine days. After this time, each tray containing larvae and the uneaten diet plus excretions was weighed on an analytical balance (Citizen CX220, Citizen Pvt. Ltd., Hermle, Gosheim, Germany). Then, larvae were recovered from each rearing tray by washing its contents with tap water and filtering with a plastic sieve. All recovered larvae from each rearing container were dried by placing them in 450 mL plastic containers with fine dry vermiculite (RADICAL, Recsa Ambiental S.A. de C.V., Tlalnepantla, Edomex, Mexico**)**. Dry larvae without vermiculite from each rearing tray/experimental run were weighed on an analytical balance (Citizen CX 220). The rearing trays were also weighed on the same analytical balance after they were cleaned and dried. The weights of rearing trays with larvae and uneaten diet plus excretions, of larvae, and of clean and dry trays were used to estimate the diet removed in each experimental run/rearing tray. Larvae from each experimental run were placed in 150 mL plastic containers with vermiculite (larvae:vermiculite ratio of 1:2). These containers were covered with pieces of pantyhose and incubated in a laboratory at 22 ± 1 °C, 70 ± 5% RH, and a 12: 12 h L: D photoperiod. Pupae were recovered from these trays 24 h after larval recovery. Pupae were maintained in a laboratory at 27 ± 1 °C, 63 ± 5% RH, and with a photoperiod of 12: 12 h L: D. A sample of pupae (ranging from two (the minimum number of pupae obtained in some rearing trays) to 104 pupae) from each experimental run was weighed after three days of the first pupal recovery. When pupae reached 12 days of age, a sample of pupae (ranging from 21 to 103 pupae) from each experimental run was used for the emergence and flight ability tests (details follow). When the experimental runs had less than 21 pupae, the pupae were used for nutritional analysis.

Macronutrient (i.e., carbohydrate, lipid, and protein) content was determined in the diets, third-instar larvae, larval excretions, and newly emerged adults. Nitrogen end products (i.e., ammonia and uric acid) were estimated in larval excretions. The general procedure consisted of three phases: sample collection and processing, carbohydrate and lipid extraction, and macronutrient and nitrogen end product determination.

#### 2.5.1. Evaluation of Response Variables

- Firmness of diets (kgF). Gel diet samples (3.5 cm diameter and 0.5 cm thickness) were prepared as indicated in Section 2.3.1. Preparation of Diets. And individually placed in 8.5 cm diameter Petri dishes. The diet samples were compressed to 1 mm (ca. 20% of its thickness) by applying 0.1 kgF pressure with a 4 cm diameter compression plate adapted to a probe of the Fruit Texture Analyzer model GS25 (Geneq Inc., Montreal, QC, Canada) adjusted to the following conditions: (i) operating range of 50–25,000 g, (ii) forward speed: 10 mm/s, (iii) reverse speed: 10 mm/s, (iv) measure speed: 10 mm/s, (v) measure distance: 1.0 mm, and (vi) reverse increment: 12 mm. Each sample was tested two to five times (depending on the available sampling points that had no evident mechanical damage and showed consistency with the readings in the same sample). To gain insights into the firmness conditions of the natural diets of *A. ludens* larvae, we also measured the firmness of a commercial host mango cv. ‘Manila’ (*M. indica*) (n = 6), a new potential host apple cv. ‘Golden Delicious’ (*M. × domestica*) (n = 4), and the wild host white sapote (*Casimiroa edulis* Llave et Lex. (Rutaceae)) (n = 4). White sapote fruit was collected at Xalapa, Veracruz, Mexico (19°32’24.15” N, 96°54’21.83” W, 1397 m elevation), mango cv. ‘Manila’ fruit was collected at Actopan, Veracruz, Mexico (19°26’31.89” N, 96°30’8.10” W, 103 m elevation), and apple cv. ‘Golden Delicious’ was purchased at Xalapa’s supply center. In the case of white sapote and mango cv. ‘Manila’, fruits were bagged with terylene cloth bags before collection to avoid natural fruit fly infestation. All the fruits analyzed were at a green-mature ripening stage, i.e., when the fruit is fully developed physically but is still green and when it is cut from the tree it does not stop ripening but rather accelerates maturation. This is the stage at which first-instar *A. ludens* larvae are found infesting such fruit in nature. From each fruit, three pieces of pulp (squares of 4 × 4 cm and 5 mm thick) from the top, middle, and bottom sections were collected with a cutter and analyzed five times with the Fruit Texture Analyzer following the same configuration conditions used for gel diet analyses.
- Penetration resistance of diets (kgF). The same samples of gel diets used for the firmness analyses were used for penetration resistance analyses. The diet samples were penetrated up to 2.5 mm (50% of the thickness of the gel-diet sample) by applying 0.1 kgF pressure with an 8 mm diameter probe attached to the Fruit Texture Analyzer adjusted to the conditions used to measure firmness. Each sample was tested one to five times in different places. Pulp from mango cv. ‘Manila’ (n = 6), apple cv. ‘Golden Delicious’ (*M. × domestica*) (n = 4), and white sapote (n = 4) were also analyzed using the same fruit as in the firmness analyses.
- pH of diets. Samples of 6 g of each gel diet were manually mixed with 5 mL of distilled water in 15 mL conical Falcon tubes. The pH was measured using a pH meter (HANNA HI2221, Hanna Instruments Inc., Woonsocket, RI, USA). Pulps from mango cv. ‘Manila’ (n = 4), apple cv. ‘Golden Delicious’ (*M. × domestica*) (n = 4), and white sapote (n = 4) were also analyzed.
- Carbohydrate, lipid, and protein contents of diets. Samples of 50 mg of each diet were diluted and manually homogenized in 500 µL of distilled water. Lipids and carbohydrates were extracted based on methods described in Warburg and Yuval [57]. In short, 100 µL of 20% sodium sulfate and 1 mL of a 1:2 ratio chloroform:methanol mixture were added to 300 µL of the diet samples. The samples were individually homogenized in a vortex (Vortex-Gene 2 G560, Scientific Industries, Bohemia, NY, USA) for ca. 1 min and centrifuged at 9402× *g* for 10 min at 4 °C in a microcentrifuge (Prism R, Labnet, Edison, NJ, USA). Then, the organic and aqueous phases were separated. For lipid extraction, the organic phase was evaporated in a heating block (Multi-Blok Heater; Lab-Line Instruments Inc., Melrose Park, IL, USA) at 80 °C for ca. 1 h. For carbohydrate extraction, the aqueous phase was evaporated in the heating block at 75 °C for ca. 3 h. The contents of carbohydrates and lipids were determined as described by Nestel et al. [58]. In short, the evaporated lipid samples obtained were diluted with 300 µL of sulfuric acid and incubated at 100 °C for 10 min. Then, 30 µL of this solution were incubated with 270 µL of vanillin (600 mg of vanillin (Sigma-Aldrich) dissolved in 100 mL of distilled water and 400 mL of 85% of H_3_PO_4_) at room temperature for 25 min. The absorbance was read at 490 nm by using a UV-Vis spectrophotometer (BioTek™ Epoch 2 Microplate Spectrophotometer, Biotek^®^ Instruments Inc., Winooski, VT, USA); triolein (Sigma-Aldrich) was used as standard. Evaporated carbohydrate samples were diluted with 200 µL of distilled water, then 50–100 µL of this solution were incubated with 1 mL of Anthron (300 mg of Anthron (Sigma-Aldrich) dissolved in 100 mL of sulfuric acid) at 90 °C for 10 min; the absorbance was monitored at 630 nm and glucose (Sigma-Aldrich) was used as standard. For protein content determination, the bicinchoninic acid (BCA) method (BCA assay kit, Sigma-Aldrich) was used with samples (2 to 5 µL depending on the opacity of the sample) of the entire homogenates, following the manufacturer’s instructions. Bovine serum albumin (BSA) was used as standard, and absorbance was read at 540 nm. Concentration curves were prepared with six concentrations of the corresponding standard in a range of 2.5–40 mg/mL for carbohydrates, 25–300 mg/mL for lipids, and 2.5–40 mg/mL for proteins, and a linear regression was adjusted to the data with an R^2^ ≥ 0.98. Then, we calculated the mg of macronutrients per mg of fresh weight considering the amount (mg) of sample used in each determination. Finally, the percentage of macronutrients per sample was calculated considering 100% at 100 mg of sample.
- Number of larvae recovered per rearing tray. The weight of all the larvae in each rearing tray was obtained and then a sample of 50 larvae was weighed. Then, the number of larvae was obtained by multiplying the weight (g) of all larvae recovered per rearing tray by 50, and the resulting product was divided by the weight (g) of 50 larvae from the same rearing tray. When the number of larvae per rearing tray was less than 50, the larvae were counted individually. The number of larvae recovered per rearing tray was the sum of the larvae sampled for the determination of nutrients and nitrogen products in larval excretions, as explained in Section 2.5. Experimental Procedures, and the estimates of the total number of larvae recovered.
- Pupation after 24 h (proportion). Estimated as the quotient of the number of pupae recovered from each diet 24 h after larval separation from the diet divided by the number of all larvae recovered from the same rearing tray.
- Pupal weight (mg). Estimated as the mean weight from samples of 2–104 three-day-old pupae; two being the minimum number of pupae obtained per rearing tray.
- Fliers (proportion). Twelve-day-old pupae were placed inside a 9 cm diameter by 10 cm high black PVC cylinder that was covered inside with a neutral talcum powder to prevent non-flying adults from escaping the cylinder by climbing. A cardboard ring was placed around the pupae at the bottom of each cylinder so that the emerging flies had a place to hang on and fly. PVC cylinders with pupae were placed in 60 cm wide by 60 cm long by 90 cm high white mesh cages containing four sticky traps (Trapper Max) and four plastic bottle traps baited with 150 mL of red wine (Padre Kino) hanging from the ceiling of the cage. Flying insects flew out of the PVC cylinders, and those not caught in the traps were manually removed three times a day. Sticky and red wine traps were replaced as needed. Two days after emergence finished, dead adults, empty puparia, partially emerged insects, and deformed adults remaining inside the cylinders were counted. Fliers were expressed as the proportion of flying insects in relation to the total number of adults that emerged.
- Diet removed per larva (g). For each experimental run/rearing tray, we first estimated the weight (g) of the uneaten diet plus excretions in the diet by subtracting the weight of the larvae and the clean and dry tray from the weight of the same tray with larvae and the uneaten diet after nine days of larval rearing. Then, we estimated the diet removed by the larvae in each rearing tray as the difference between the initial fresh weight (g) of the diet and the weight of the uneaten diet plus excretions minus the percentage of fresh weight loss estimated from an additional batch of the same diet formulation without larvae (as indicated in Section 2.3.1. Preparation of Diets). When the diets were collected with larvae, the larvae were removed from the diets as explained in Section 2.5. Experimental Procedures. Diet removed per larva (g) was then estimated by dividing the diet removed (g) per rearing tray by the number of larvae recovered from the same rearing tray.
- Relative consumption of carbohydrates, lipids, and proteins per larva (g). Based on the estimations of diet removed per larva and on the analyses of nutrient content in the diets, the relative consumption of macronutrients was calculated by multiplying the diet removed per larva (g) by the carbohydrate, lipid, and protein contents of the diets and dividing the product by 100.
- Carbohydrate, lipid, and protein contents in larvae and larval excretions. Seven days after larvae were inoculated into the diets, we sampled ca. 10% of the estimated number of larvae inoculated into each rearing tray (between 6 and 75 larvae per tray). The larvae were washed with purified water, dried with a paper towel, and placed in 8.5 cm diameter sterile Petri dishes at 29 ± 1 °C and 70 ± 5% RH for 24 h. Then, the larvae from each rearing tray were weighed on an analytical balance (Sartorius CP64, Sartorius AG, Goettingen, Germany), transferred to vials, frozen in liquid nitrogen, and homogenized in a mortar with a pestle by using 200 µL of sodium phosphate buffer (50 mM, pH 7.4) (PB) per larva. These samples were kept at −80 °C in a freezer (Thermo Fisher Scientific, FORMA 900 Series, Marietta, OH, USA) until analysis. To collect larval excretions, the Petri dishes in which the larvae were kept for 24 h were washed with 500 to 1000 µL of Milli-Q water. The washing water was poured into 1.5 mL vials. Samples were then dried for 12 h at 45 °C in an incubator (Binder BD 53-UL E2, GmbH, Bohemia, NY, USA). After drying, the concentrated samples were diluted in 400 µL of distilled water. For carbohydrate, lipid, and protein contents, we followed the procedure explained before for the diets.
- Ammonia and uric acid in larval excretions. An amount of 2 to 20 μL of the larval excretions, obtained as described above, were analyzed as indicated in the commercial assay kits AA0100 and MAK077 (Sigma-Aldrich, St. Louis, MO, USA).
- Carbohydrate, lipid, and protein contents in female and adult flies. Pools of five newly emerged (less than 12 h) females and five males were weighed, frozen, and homogenized with 1 mL of PB in a Tissue Lyser II (Qiagen^®^, Hilden, Germany) at 30 rpm for 3 min. The carbohydrate, lipid, and protein contents were determined as explained above.

### 2.6. Statistical Analyses

Analyses followed the procedure of the Design-Expert^®^ 10 software (StatEase Inc., Minneapolis, MN, USA) for a response surface randomized design [50]. Details on the statistical procedures can be found in the report by Pascacio-Villafán et al. [25,32]. In short, polynomial models from the mean to the fifth order were fitted sequentially to the values of each response variable (Table 2) as a function of the explanatory variables (i.e., gel type, gel content in the diet, larval density in the diet, and their double interactions). Model selection was then based on: (i) a sequential model sum of squares (Type I) to assess if the addition of new terms improved the explanatory power of the model, (ii) a lack-of-fit test, and (iii) adjusted and predicted R^2^ values [50]. The selected model was then evaluated by analysis of variance (classical sum of squares—Type II) after backward elimination of non-significant model terms based on a corrected Akaike information criterion (AICc) [50]. After model fitting, the normal distribution and equal variance of residuals were checked graphically. Graphical Box–Cox analyses were used to examine if a transformation could improve the model fit. The values of the following response variables were transformed: diet firmness (power, λ = 0.63), acidity (power, λ = −2.66), number of larvae recovered from diets (square root), fliers (power, λ = 3), diet removed per larva (inverse square root), relative carbohydrate consumption per larva (square root), relative lipid consumption per larva (natural log), relative protein consumption per larva (natural log), carbohydrates in larval excretions (square root), lipids in larval excretions (base 10 log), proteins in larval excretions (square root), ammonia in larval excretions (natural log), uric acid in larval excretions (natural log), lipids in larvae (square root), carbohydrates in females (square root), lipids in females (square root), and lipids in males (inverse square root). In the case of the variables diet removed per larva, and the relative consumption of carbohydrates, lipids and proteins per larva we detected three outliers and highly influential data points with unrealistic values of diet removal (i.e., between 1.5 and 3.1 g of diet removed per larva) that we believe reflect measurement errors [59]. Therefore, these data points were eliminated from the analyses. The mean estimates are reported together with the least significant difference (LSD) bars [50]. Results are presented in terms of statistical clarity [60].

## 3. Results

### 3.1. Physical, Chemical, and Nutritional Traits of the Diets

#### 3.1.1. Firmness

**Figure 2:**
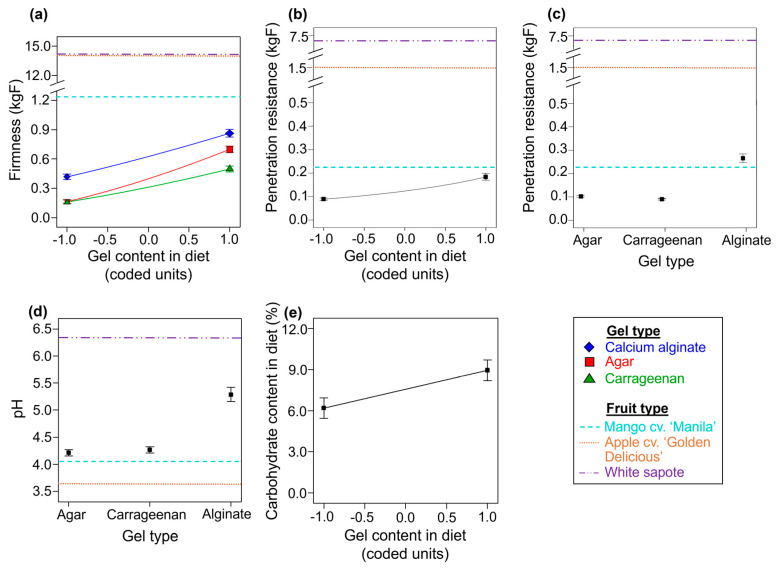
(**a**) Interaction effects between the gel type and the gel content on the firmness of diets (kgF). Main effects of (**b**) the gel content in the diet and (**c**) the gel type on the penetration resistance of diets (kgF). (**d**) Main effects of the gel type on the acidity of the diets (pH units). (**e**) Main effects of the gel content on the carbohydrate content of the diets (%). In (**a**–**d**), the horizontal lines indicate the mean values (n = 4) of the firmness, penetration resistance, and acidity of mango cv. ‘Manila’, apple cv. ‘Golden Delicious’, and white sapote pulp. In the case of the main effects models (**b**–**e**), the graphs show estimations made averaging over all the levels of the missing factor (i.e., the gel type in panels (**b**,**e**); and the gel content in the diet in panels (**c**,**d**)). In (**a**,**b**,**e**), the change in each response variable estimated by the fitted models is shown through a continuous scale of gel content in the diet, and the extreme ends of each model are accompanied by mean estimates and LSD bars to allow visual appreciation of differences between end points. In (**c**,**d**), “Alginate” is used as an abbreviation of “Calcium alginate” and error bars around mean estimates indicate the LSD between means.

A reduced two-factor interaction model was fitted to the data on diet firmness (*F* = 169.82; df = 5, 59; *p* < 0.0001; R^2^_adj_ = 0.929). We found clear statistical main effects for the gel content in the diet (*F* = 564.12; df = 1, 59; *p* < 0.0001), the gel type (*F* = 187.09; df = 2, 59; *p* < 0.0001), and their interaction (*F* = 9.37; df = 2, 59; *p* = 0.0003) on the firmness of the experimental diets. Regardless of the gel type used, diet firmness increased as the gel content in the diet increased (Figure 2a). Calcium alginate diets were firmer than agar and carrageenan diets across the range of gel contents tested (Figure 2a). The firmness of the agar and carrageenan diets was similar at lower gel content values but differed as the gel content increased, with agar having greater firmness than carrageenan (Figure 2a). The firmness of pulp from apple cv. ‘Golden Delicious’ and white sapote was higher than the firmness of mango cv. ‘Manila’ by ca. 10 times, and the firmness of the pulp of all the fruit evaluated (mango cv. ‘Manila’, apple cv. ‘Golden Delicious’, and white sapote) was higher than that of the gel diets (Figure 2a).

#### 3.1.2. Penetration Resistance

In the case of diets with agar levels below 0.0 coded units, no penetration resistance readings were obtained because the diets collapsed when penetration was attempted. In the case of diets with carrageenan, readings were only obtained for diets with gel contents of 0.5 and 1.0 coded units**.** A reduced linear model was fitted to the data on the penetration resistance of the diets (*F* = 187.25; df = 3, 28; *p* < 0.0001; R^2^_adj_ = 0.947). The model indicated clear statistical effects for the gel content in the diet (*F* = 132.41; df = 1, 28; *p* < 0.0001) and the type of gel (*F* = 278.35; df = 2, 28; *p* < 0.0001) on the penetration resistance of the diets. Regardless of the type of gelling agent used in the diets, the penetration resistance increased as the gel content in the diets increased (Figure 2b). Overall, the calcium alginate diets were more resistant to penetration than the agar and carrageenan diets (Figure 2c). The penetration resistance of the agar and carrageenan diets was very similar within the range of gel contents tested (Figure 2c). The white sapote pulp showed the highest mean value for penetration resistance, followed by the apple cv. ‘Golden Delicious’ and mango cv. ‘Manila’ pulp (Figure 2b,c). The mean penetration resistance of the calcium alginate diets was slightly higher than that of mango cv. ‘Manila’ pulp, whereas the penetration resistance of the agar and carrageenan diets was lower than that of the pulps of all the fruits analyzed (Figure 2c).

#### 3.1.3. Acidity

The best fitting model to describe diet acidity was a reduced linear model (*F* = 69.91; df = 3, 61; *p* < 0.0001; R^2^_adj_ = 0.764). We found clear statistical main effects only for the gel type on the acidity of diets (*F* = 98.23; df = 2, 61; *p* < 0.0001). The mean pH units of the agar (4.2) and carrageenan (4.3) diets were moderately acidic, whereas calcium alginate diets were less acidic, with a mean pH level of 5.3 (Figure 2d). The effect of the gel content in diets on their acidity was not statistically clear (*F* = 3.41; df = 1, 61; *p* = 0.0698). Mango cv. ‘Manila’ and apple cv. ‘Golden Delicious’ pulps were more acidic than the agar and carrageenan diets (Figure 2d). The pulp from white sapote was the least acidic among all the fruit pulp and gel diets tested, with a pH of 6.3 (Figure 2d).

#### 3.1.4. Carbohydrate, Lipid, and Protein Contents of Diets

- Carbohydrates: A reduced linear model was fitted to describe the carbohydrate content in the diets as a function of the gel type and the gel content in diets (*F* = 5.86; df = 3, 61; *p* = 0.0014; R^2^_adj_ = 0.185). The carbohydrate content of diets increased as the gel content in diets increased (*F* = 13.46; df = 1, 61; *p* = 0.0005; Figure 2e). The effect of the gel type on the carbohydrate content of diets was statistically unclear (*F* = 3.13; df = 2, 61; *p* = 0.0509).
- Lipids and proteins: A reduced two-factor interaction model indicated that the effects of the gel type and the gel content in the diets on the lipid content were not statistically clear (*F* = 1.44; df = 5, 59; *p* = 0.2239; R^2^_adj_ = 0.033). In the case of the protein content, the best descriptor of the data was a null model with an overall mean (std. dev.) of 3.39% (1.78%).

### 3.2. Production and Quality Parameters of Flies

#### 3.2.1. Estimated Number of Larvae Recovered from Diets

**Figure 3:**
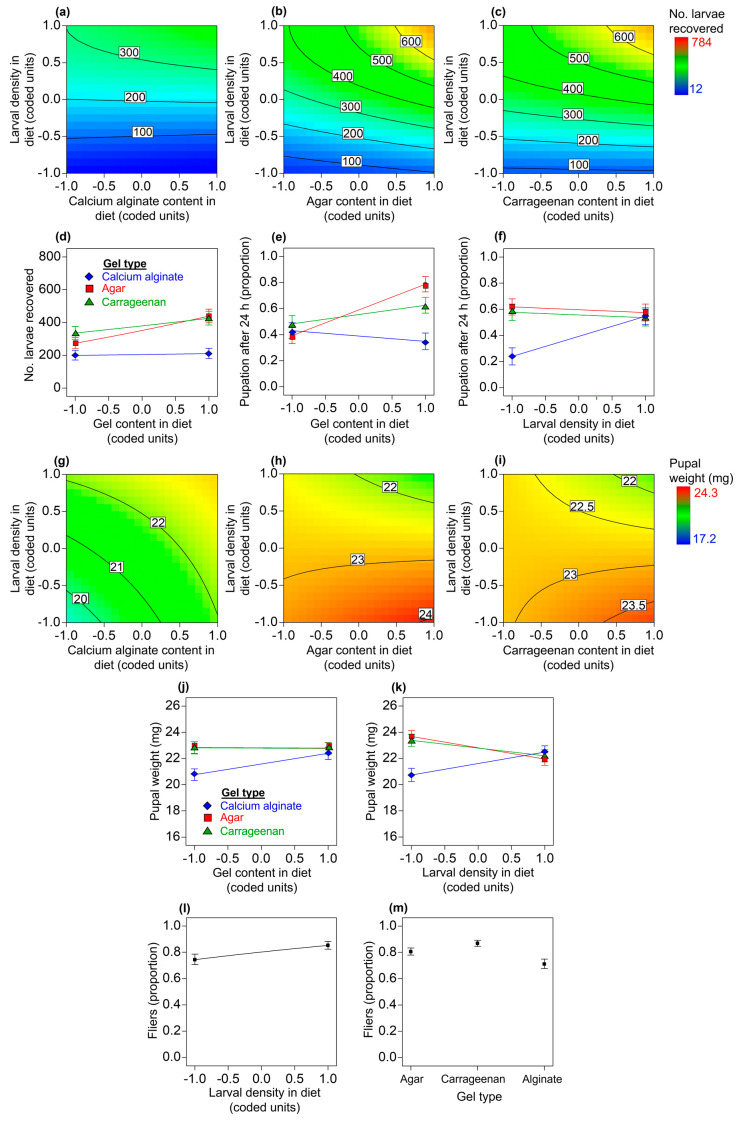
(**a**–**c**) Response surfaces of the estimated number of larvae recovered from calcium alginate, agar, and carrageenan diets, explained by the interaction effects of the gel content and the larval density in diets. (**d**) Interaction effects between the gel type and the gel content in the diet on the estimated number of larvae recovered from diets. (**e**,**f**) Interaction effects between the gel content in the diet and the gel type and between the larval density in the diet and the gel type, respectively, on pupation after 24 h (proportion). (**g**–**i**) Response surfaces of pupal weight (mg) of flies reared on calcium alginate, agar, and carrageenan diets, explained by the interaction effects of the gel content and the larval density in the diet. (**j**,**k**) interaction effects between the gel type and the gel content and between the gel type and the larval density in diet, respectively, on pupal weight (mg). (**l**,**m**) Main effects of the larval density in diet and the gel type on the proportion of fliers. In (**a**–**c**,**g**–**i**) the color scale indicates the overall range of experimental values recorded. In the case of the main effects models (**l**,**m**), the graphs show estimations made averaging over all the levels of the missing factors (i.e., the gel type and the gel content in the diet in panel **l** and the gel content and the larval density in the diet in panel (**m**)). In (**d**–**f**,**j**,**k**), the graphs show estimations made averaging over all the levels of the larval density (**d**,**e**,**j**) and the gel content in the diet (**f**,**k**). In (**d**–**f**,**j**–**l**), the change in each response variable estimated by the fitted models is shown through a continuous scale of the explanatory variable, and the extreme ends of each model are accompanied by mean estimates and LSD bars to allow visual appreciation of differences between end points. In (**m**), “Alginate” is used as an abbreviation of “Calcium alginate” and error bars around the mean estimates indicate the LSD between means.

A reduced quadratic model was used to explain the effects of the predictors on the estimated number of larvae recovered from the diets (*F* = 71.71; df = 8, 56; *p* < 0.0001; R^2^_adj_ = 0.898). We found main and interaction statistical effects for the explanatory variables on the estimated number of larvae recovered from the diets (gel content in diet: *F* = 13.76; df = 1, 56; *p* = 0.0005; larval density in diet: *F* = 449.18; df = 1, 56; *p* < 0.0001; gel type: *F* = 45.35; df = 2, 56; *p* < 0.0001; gel content in diet × larval density in diet: *F* = 5.16; df = 1, 56; *p* = 0.0270; gel content in diet × gel type: *F* = 3.19; df = 2, 56; *p* = 0.0487; larval density in diet^2^: *F* = 27.91; df = 1, 56; *p* < 0.0001). Overall, more larvae were recovered from the agar and carrageenan diets than from the calcium alginate diets (Figure 3a–d). We found a positive effect for larval density on the number of larvae recovered from diets, with curvature towards the highest levels of gel content in the diet (Figure 3a–c). In the calcium alginate diets, the effect of the gel content in the diet on the number of larvae recovered from the diets was negligible, but in the agar and carrageenan diets, the number of larvae recovered increased as the gel content in the diet rose (Figure 3d).

#### 3.2.2. Pupation after 24 h

The best-fit model to describe the pupation of flies 24 h after larval separation from the diet was a two-factor interaction model (*F* = 12.29; df = 8, 56; *p* < 0.0001; R^2^_adj_ = 0.585). The model indicated clear statistical main effects for the gel content in the diet (*F* = 18.55; df = 1, 56; *p* < 0.0001) and the gel type (*F* = 16.21; df = 2, 56; *p* < 0.0001) and interaction effects between the gel content in the diet and the gel type (*F* = 12.58; df = 2, 56; *p* < 0.0001) and between the larval density in the diet and the gel type (*F* = 7.67; df = 2, 56; *p* = 0.0011) on pupation of flies. At low levels of dietary gel content, pupation was similar among flies from the different diets; however, pupation diverged as the gel content in the diets increased—it increased linearly as the gel content in the diet increased for agar and carrageenan diets but decreased for calcium alginate diets (Figure 3e). Larval density positively affected pupation only in the calcium alginate diets (Figure 3f).

#### 3.2.3. Pupal Weight

A reduced two-factor interaction model was fitted to the data on pupal weight (*F* = 9.05; df = 9, 54; *p* < 0.0001; R^2^_adj_ = 0.535). We found clear statistical main effects for the larval density (*F* = 4.14; df = 1, 54; *p* = 0.0469) and the gel type (*F* = 14; df = 2, 54; *p* < 0.0001) and interaction effects between the gel content in the diet and the larval density in the diet (*F* = 5.87; df = 1, 54; *p* = 0.0188), the gel content in the diet and the gel type (*F* = 3.61; df = 2, 54; *p* = 0.0337), and the larval density in the diet and the gel type (*F* = 11.29; df = 2, 54; *p* < 0.0001) on pupal weight. The lowest pupal weights were observed in flies reared on the calcium alginate diets (Figure 3g), whereas the heaviest pupae were found when using agar or carrageenan diets (Figure 3h,i). The highest pupal weights estimated for calcium alginate diets were with high levels for both the gel content and the larval density (Figure 3g), whereas for the agar and carrageenan diets the highest pupal weights were estimated in diets with high levels of gel content and low larval densities (Figure 3h,i). Pupal weight increased with gel content only in the case of calcium alginate diets (Figure 3j). Overall, the pupae of flies from the agar and carrageenan diets showed a slight decrease in weight as the larval density increased; however, in the case of pupae from the calcium alginate diets, the pupal weight increased with larval density (Figure 3k).

#### 3.2.4. Fliers

A reduced linear model was fitted to the data on flying insects (*F* = 12.1; df = 3, 57; *p* < 0.0001; R^2^_adj_ = 0.357). We found clear statistical main effects of the larval density in the diet (*F* = 10.43; df = 1, 57; *p* = 0.0021) and the gel type (*F* = 15.26; df = 2, 57; *p* < 0.0001) on the emergence of flying insects. Regardless of the gel type, the proportion of fliers increased with the larval density in the diet (Figure 3l), but the mean proportion of fliers from calcium alginate diets was clearly lower than the proportion of fliers from the agar or carrageenan diets (Figure 3m).

### 3.3. Diet Removal and Larval and Adult Nutritional Traits

#### 3.3.1. Diet Removed per Larva

**Figure 4:**
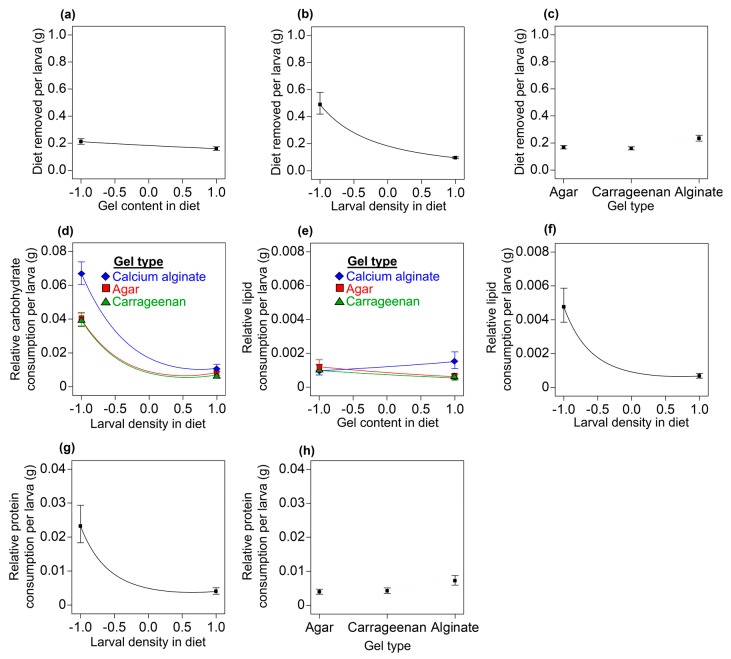
Main effects of (**a**) the gel content in the diet, (**b**) the larval density in the diet, and (**c**) the gel type on diet removed per larva (g). (**d**) Interaction effects between the gel type and the larval density in the diet on relative carbohydrate consumption per larva (g). (**e**) Interaction effects between the gel type and the gel content in the diet on relative lipid consumption per larva. (**f**) Main effects of the larval density in the diet on relative lipid consumption per larva. Main effects of (**g**) the larval density in the diet and (**h**) the gel type on relative protein consumption per larva (g). In the case of the main effects models, the graphs show estimations made by averaging over all the levels of the missing factors (i.e., the gel type and the larval density in the diet in panel **a**, the gel type and the gel content in the diet in panels (**b**,**f**,**g**), and the gel content and the larval density in the diet in panels (**c**,**h**)); in the case of the two-factor interaction models, the graphs show estimations made by averaging over all the levels of the gel content in the diet in (**d**) and the larval density in the diet in (**e**). In (**a**,**b**,**d**–**g**), the change in each response variable estimated by the fitted models is shown through a continuous scale of the explanatory variable and the extreme ends of each model are accompanied by mean estimates and LSD bars to allow visual appreciation of differences between end points. In (**c**,**h**), “Alginate” is used as an abbreviation of “Calcium alginate” and error bars around mean estimates indicate the LSD between means.

Diet removed per larva: A linear model was fitted to describe the diet removed per larva (*F* = 69.88; df = 4, 57; *p* < 0.0001; R^2^_adj_ = 0.819). We found clear statistical main effects for the gel content in the diet (*F* = 9.72; df = 1, 57; *p* = 0.0029), the larval density in the diet (*F* = 252.18; df = 1, 57; *p* < 0.0001), and the gel type (*F* = 12.33; df = 2, 57; *p* < 0.0001) on the estimated amount of diet removed per larva. Less of the diet was removed per larva as the gel content in the diets increased (Figure 4a). An increased larval density also resulted in a reduction in the amount of diet removed per larva (Figure 4b). More diet was removed by larvae in the calcium alginate diets than in the agar or carrageenan diets (Figure 4c).

#### 3.3.2. Relative Consumption of Carbohydrates, Lipids, and Proteins per Larva

- Carbohydrates: A reduced quadratic model was fitted to the data on relative carbohydrate consumption per recovered larvae (*F* = 60.74; df = 6, 55; *p* < 0.0001; R^2^_adj_ = 0.855). The model indicated clear statistical main effects for the larval density in the diet (*F* = 2.76; df = 1, 55; *p* < 0.0001), the gel type (*F* = 24.75; df = 2, 55; *p* < 0.0001), and the larval density in the diet × the gel type (*F* = 3.29; df = 2, 55; *p* = 0.0447) and a quadratic effect for larval density in the diet (*F* = 80.34; df = 1, 55; *p* < 0.0001) on the relative amount of carbohydrate consumed per larva. A higher relative consumption of carbohydrates per larva was observed in calcium alginate diets at low larval densities when compared with the agar and carrageenan diets; however, at high larval densities the relative amount of carbohydrates consumed per larva decreased and did not differ among diets (Figure 4d).
- Lipids: A reduced quadratic model was used to describe the relative consumption of lipid per recovered larvae (*F* = 15.03; df = 7, 54; *p* < 0.0001; R^2^_adj_ = 0.617). The model showed clear statistical main effects for the larval density in the diet (*F* = 78.2; df = 1, 54; *p* < 0.0001) and the gel type (*F* = 3.76; df = 2, 54; *p* = 0.0295) and quadratic effects for larval density in the diet (*F* = 15.66; df = 1, 54; *p* = 0.0002) on relative lipid consumption. However, the main effects of the gel content in the diet (*F* = 3.46; df = 1, 54; *p* = 0.0684) and the interaction effects of the gel content in the diet × gel type (*F* = 3.16; df = 2, 54; *p* = 0.0504) were not statistically clear. Relative lipid consumption per larva decreased quadratically as the larval density in the diet increased (Figure 4e). A trend towards higher relative lipid intake was observed in calcium alginate diets with high gel contents compared with agar and carrageenan diets (Figure 4f).
- Proteins: A reduced quadratic model was used to describe the relative protein consumption per larva (*F* = 15.73; df = 4, 57; *p* < 0.0001; R^2^_adj_ = 0.491). We found clear statistical main effects for the larval density in the diet (*F* = 48.47; df = 1, 57; *p* < 0.0001) and the gel type (*F* = 5.58; df = 2, 57; *p* = 0.0061) and quadratic effects for larval density in the diet (*F* = 12.58; df = 1, 57; *p* = 0.008) on the relative protein intake by individual larva. We found a curved decrease in the relative quantity of proteins consumed per larva as larval density in the diet increased (Figure 4g). We found a higher relative consumption of proteins in larvae reared on the calcium alginate diets than on the agar and carrageenan diets (Figure 4h).

#### 3.3.3. Carbohydrates, Lipids, and Proteins in Excretions of Third-Instar Larvae

**Figure 5:**
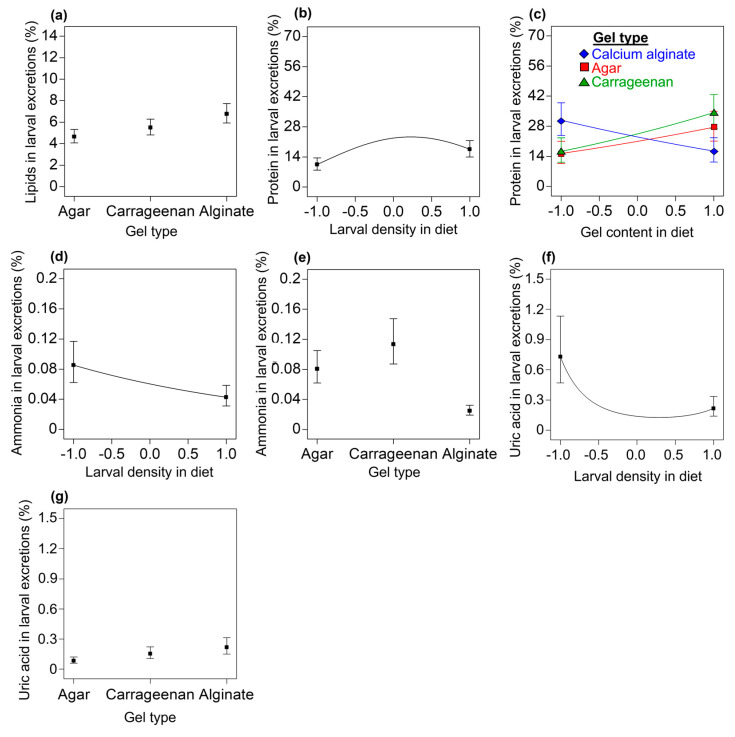
(**a**) Main effects of the gel type on lipids in larval excretions (%). (**b**) Main effects for the larval density in the diet on protein in larval excretions (%). (**c**) Interaction effects between the gel type and the gel content in the diet on protein in larval excretions (%). (**d**) Main effects for the larval density and (**e**) gel type on the ammonia in larval excretions (%). (**f**) Main effects for the larval density and (**g**) gel type on the uric acid in larval excretions (%). In the case of main effects models, the graphs show estimations made by averaging over all the levels of the missing factors (i.e., the gel content and the larval density in the diet in panels (**a**,**e**,**g**); and the gel type and the gel content in the diet in panels (**b**,**d**,**f**)). In (**c**), graphs show estimations made averaging over all the levels of the larval density in the diet. In (**b**–**d**,**f**), the change in each response variable estimated by the fitted models is shown through a continuous scale of the explanatory variable and the extreme ends of each model are accompanied by mean estimates and LSD bars to allow visual appreciation of differences between end points. In (**a**,**e**,**g**) “Alginate” is used as an abbreviation of “Calcium alginate” and error bars around the mean estimates indicate the LSD between means.

- Carbohydrates: A null model indicated that, regardless of the gel type, for the gel content and the larval density treatments an overall mean (std. dev.) of 4.34% (0.57%) provided the best description of the carbohydrate content in larval excretions.
- Lipids: A reduced linear model indicated that the gel type used had a clear statistical effect on the percentage of lipids found in larval excretions, with a higher mean percentage of lipids found in excretions from larvae reared on the calcium alginate diets (*F* = 3.92; df = 2, 62; *p* = 0.0250; R^2^_adj_ = 0.084; Figure 5a).
- Proteins: A reduced quadratic model was fitted to the data on the percentage of protein in larval excretions (*F* = 3.83; df = 7, 57; *p* = 0.0018; R^2^_adj_ = 0.236). We found a clear statistical relationship between the protein content in larval excretions and the larval density in the diet (linear effect: *F* = 4.37; df = 1, 57; *p* = 0.0411; quadratic effect: *F* = 8.17; df = 1, 57; *p* = 0.0059), with an initial positive effect as the larval density increased to ca. intermediate larval densities that remained somewhat stable until approaching the highest larval densities and then began to decrease (Figure 5b). We also found clear statistical effects for an interaction between the gel type and the gel content in the diet (*F* = 5.34; df = 2, 57; *p* = 0.0075) affecting the protein content in larval excretions. At low levels of gel content in the diets, the excretions of larvae from the calcium alginate diets had more protein than the excretions of larvae from the agar and carrageenan diets; however, the protein percentage in excretions from larvae on the calcium alginate diets decreased as the gel content in the diet increased, whereas the protein content in the excretions of larvae from the agar and carrageenan diets showed an opposite trend (Figure 5c).

#### 3.3.4. Ammonia and Uric Acid in the Excretions of Third-Instar Larvae

- Ammonia: A reduced linear model was fitted to the data on the percentage of ammonia in the excretions of third-instar *A. ludens* larvae (*F* = 14.56; df = 3, 61; *p* < 0.0001; R^2^_adj_ = 0.388). The ammonia content in larval excretions decreased as the larval density increased (*F* = 4.81; df = 1, 61; *p* = 0.032; Figure 5d), and larvae reared on the calcium alginate diets had the lowest levels of ammonia in their excretions when compared with the excretions of larvae from the agar and carrageenan diets (*F* = 18.36; df = 2, 61; *p* < 0.0001; Figure 5e).
- Uric acid: A reduced quadratic model was fitted to the data on the percentage of uric acid in the excretions of third-instar *A. ludens* larvae (*F* = 4.00; df = 6, 58; *p* = 0.002; R^2^_adj_ = 0.220). We found a clear statistical relationship between the uric acid levels in larval excretions and the larval density in the diet (linear effect: *F* = 8.28; df = 1, 58; *p* = 0.0056; quadratic effect: *F* = 8.56; df = 1, 58; *p* = 0.0049). The content of uric acid in larval excretions decreased as larval density increased, from an estimated 0.73% at the lowest larval densities to 0.22% at the highest larval density (Figure 5f). Larvae from the calcium alginate diets had the highest levels of uric acid in their excretions, followed by larvae from the carrageenan diets (*F* = 3.42; df = 2, 58; *p* = 0.0393; Figure 5g). The main effects for the gel content in the diet (*F* = 0.46; df = 1, 58; *p* = 0.4980) and the interaction effects between the gel content in the diet and the larval density in the diet (*F* = 2.41; df = 1, 58; *p* = 0.1257) were not statistically clear.

#### 3.3.5. Carbohydrates, Lipids, and Proteins in Third-Instar Larvae

**Figure 6:**
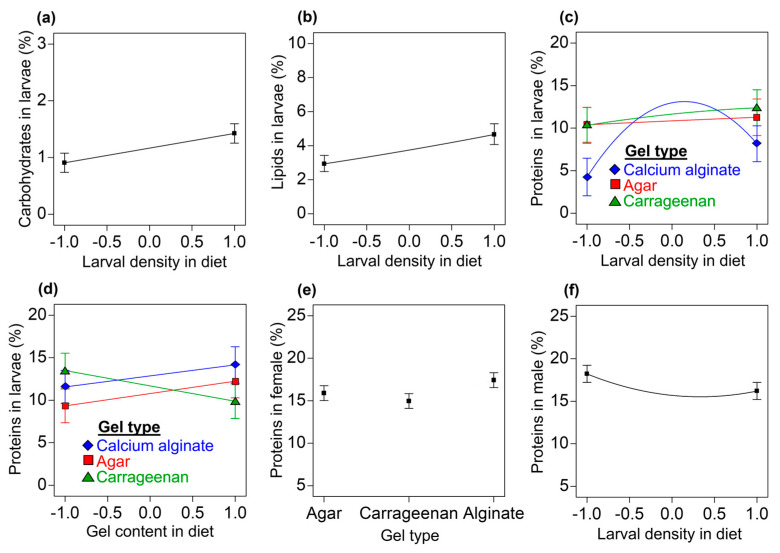
(**a**) Main effects for the larval density in the diet on carbohydrates in larvae (%). (**b**) Main effects for the larval density in the diet on lipids in the larvae (%). (**c**) Interaction effects between the gel type and the larval density in the diet and (**d**) between the gel type and the gel content in the diet on proteins in larvae (%). Main effects for (**e**) the gel type on protein levels in adult females and (**f**) the larval density in the diet on protein levels in adult males (%). In (**a**,**b**,**f**), graphs show estimations made by averaging over all the levels of the gel type and the gel content in the diet; in (**c**,**d**), graphs show estimations made by averaging over all the levels of the gel content in the diet and the larval density in the diet, respectively; in (**e**), estimations were made by averaging over all the levels of gel content and larval density in the diet. In (**a**–**d**,**f**), the change in each response variable estimated by the fitted models is shown through a continuous scale of the explanatory variable and the extreme ends of each model are accompanied by mean estimates and LSD bars to allow visual appreciation of differences between end points. In (**e**), “Alginate” is used as an abbreviation of “Calcium alginate” and error bars around the mean estimates indicate the LSD between means.

- Carbohydrates: The carbohydrate content in third-instar *A. ludens* larvae increased as larval density in the diet increased (reduced linear model: *F* = 9.24; df = 1, 48; *p* = 0.0034; R^2^_adj_ = 0.114; Figure 6a).
- Lipids: A reduced linear model was fitted to the data on the lipid content in larvae (*F* = 4.76; df = 3, 61; *p* = 0.0048; R^2^_adj_ = 0.150). The lipid content in larvae increased as the larval density in the diet increased (*F* = 10.12; df = 1, 61; *p* = 0.0023; Figure 6b). The effect of the gel type used in diets on the lipid content in larvae was statistically unclear (*F* = 2.61; df = 2, 61; *p* = 0.0816).
- Proteins: A reduced cubic model was fitted to the data on the protein content in larvae (*F* = 2.49; df = 11, 53; *p* = 0.0132; R^2^_adj_ = 0.204). We found clear statistical quadratic effects for the larval density in the diet (*F* = 5.08; df = 1, 53; *p* = 0.0284) and interaction effects between the quadratic larval density term and the gel type (*F* = 5.05; df = 2, 53; *p* = 0.0099) on the larval protein content. The protein content of larvae from the agar and carrageenan diets showed a slight increase as larval density increased (Figure 6c), whereas for larvae from the calcium alginate diets, their initial protein content was lower than that observed in the agar and carrageenan diets but it increased notably as the larval density increased up to ca. intermediate densities, after which the protein content in the larvae began to decrease (Figure 6c). We also found clear statistical interactions between the gel type and the gel content in the diet (*F* = 3.45; df = 2, 53; *p* = 0.0391). The protein content in larvae from diets with the lowest level of carrageenan was higher than in larvae from the agar and calcium alginate diets; however, at the highest gel contents, the highest protein contents in larvae were observed in flies reared on calcium alginate diets (Figure 6d). The linear effects of the gel content in the diet (*F* = 0.52; df = 1, 53; *p* = 0.4740), the larval density in the diet (*F* = 3.09; df = 1, 53; *p* = 0.0848), and the gel type (*F* = 1.2; df = 2, 53; *p* = 0.6394) were statistically unclear, as was the interaction between the linear terms of larval density in the diet and the gel type (*F* = 0.45; df = 2, 53; *p* = 0.6394).

#### 3.3.6. Carbohydrates, Lipids, and Proteins in Adult Female and Male Flies

- Carbohydrates: The carbohydrate content in female flies was better explained by a null model with an overall mean (std. dev.) of 0.705% (0.04%). In the case of male flies, a reduced quartic model showed no clear statistical effect for the predictors on the carbohydrate content of males (*F* = 1.17; df = 17, 46; *p* = 0.3247; R^2^_adj_ = 0.044).
- Lipids: The effects of the predictors on the lipid content in female flies were statistically unclear (reduced linear model: *F* = 3.02; df = 2, 61; *p* = 0.0563; R^2^_adj_ = 0.060). Likewise, the effects of the predictors on the lipid content in male flies were statistically unclear (reduced quartic model: *F* = 1.59; df = 20, 43; *p* = 0.0997; R^2^_adj_ = 0.158).
- Proteins: The gel type had clear statistical effects on the protein content in female flies (reduced linear model: *F* = 4.09; df = 2, 61; *p* = 0.0215; R^2^_adj_ = 0.090). Females from calcium alginate diets had, on average, 1.5 and 2.5% more protein content than females from the agar and carrageenan diets, respectively (Figure 6e). A reduced quadratic model was fitted to the data on protein content in adult males (*F* = 3.48; df = 2, 61; *p* = 0.0370; R^2^_adj_ = 0.073). The model indicated that the main (*F* = 3.97; df = 1, 61; *p* = 0.0507) and quadratic (*F* = 3.36; df = 1, 61; *p* = 0.0716) effects of the larval density on the protein content in male flies were statistically unclear (Figure 6f).

The response variables modeled by RSM are presented in Table S1. The means and standard errors of each response variable for each of the gel types used in diets (without considering the effects of the gel content and larval density in the diet) are found in Table S2.

## 4. Discussion

Our experimental and statistical modeling work provides evidence in favor of agar and carrageenan gel diets over calcium alginate gel diets for rearing the economically important tephritid fly pest *A. ludens*. Contrary to our working hypothesis that calcium alginate was as good a gelling agent as either agar or carrageenan in the *A. ludens* larval diet, we found that the number of larvae recovered, the pupation percentage, the pupal weight, and the proportion of fliers were lower in calcium alginate diets compared with the agar and carrageenan diets (Figure 3). We show that the firmness and resistance to penetration of the diets increased with the level of the gel content and that, overall, the calcium alginate diets were firmer, more resistant to penetration, and less acidic than the agar and carrageenan diets (Figure 2a–d). The agar and carrageenan diets and the flies from these diets were very similar to each other but differed markedly from the calcium alginate diets and flies from those diets for most of the response variables evaluated (Figure 2, Figure 3, Figure 4, Figure 5 and Figure 6), except for larval protein content, where we found a mild decrease in the protein content as the carrageenan content in the diet increased. The differences in the firmness, penetration resistance, and acidity characteristics between the calcium alginate diets and the agar and carrageenan diets could help to explain why the calcium alginate diets produced fewer high-quality *A. ludens* individuals than the agar and carrageenan diets (Figure 3). An extremely firm medium that is resistant to penetration, such as the calcium alginate diets in this study (Figure 2a,b), may limit the movement of larvae tunneling inside the diet and their ability to suck and swallow the food [15]. In such a situation, the larvae could experience a negative flow of energy (i.e., the energy required to move and feed in an extremely firm and resistant to penetration medium is higher than the energy acquired from the food), with negative effects on fly development, functioning, and survival [53]. This could partially explain why we recovered lower numbers of larvae from the calcium alginate diets than from the agar and carrageenan diets (Figure 3a–c). The idea that extremely high firmness and penetration resistance characteristics of diets pose a physical barrier to optimal larval feeding could also help explain why we found a reduction in diet removal by larvae as the gel content in the diets increased (Figure 4a). On the other hand, the reduction in the amount of diet removed and relative nutrient consumption per larva as the larval density increased (Figure 4b,d,e,g,) appears like a predictable result considering that food resources were limited and the higher the number of individuals, the lower the food resources available for each of them. Our results support those of previous studies highlighting the relationship between the physicochemical characteristics of the larval diet and the nutrition and life history traits of flies [17,28,29].

The potentially adverse conditions of an extremely firm and resistant to penetration medium might be overcome by the cooperative feeding behavior of large groups of larvae [32,34]. In fact, we found that extremely high larval densities in calcium alginate diets (which were the firmest and more resistant to penetration diets, Figure 2a,c) had a positive effect on the proportion of flies that pupated (Figure 3f) and on their pupal weights (Figure 3k) compared with flies developed under extremely low larval density treatments. The pupation and pupal weight of tephritid flies are influenced by the lipid and protein reserves acquired during the larval stage [53,58]. Here, we show that regardless of the gel type and its content in the diets, the lipid and carbohydrate content in third-instar *A. ludens* larvae increased with the levels of the larval density treatment (Figure 6a,b). This might be related to the ability of groups of *A. ludens* larvae to produce extraoral digestion secretions that facilitate feeding and improve larval nutrition [34]. The effects of larval density on the protein content in third-instar larvae reared on the calcium alginate diets resembled the ‘‘inverted-U’’ dose–response curve from the toxicological concept of hormesis [61], a pattern that was not observed in larvae reared on the agar and carrageenan diets (Figure 6c). This suggests that in *A. ludens* reared on very firm, penetration-resistant media, low to medium larval densities favor protein assimilation, whereas extremely high larval densities inhibit it. Another possible explanation for the increase in larval protein content as larval density increased could be attributed to the exuviae of larvae providing a source of food biomass; however, this needs to be explored in future studies. On the other hand, we found that (regardless of the type of gel) increased larval densities caused a quadratic increase in the protein content in third-instar larval excretions (Figure 5b) and a decrease in the nitrogenous products derived from protein metabolism (Figure 5d,f). This suggests that a portion of the protein ingested with the diet was not used by third-instar larvae, passing through their guts without being metabolized and assimilated by the insect. Future studies should investigate in detail the nutrition of *A. ludens* larvae across their different developmental stages. A deeper understanding of larval nutrition will allow the development of more cost-effective artificial diets if the precise level of protein that the larvae need for optimal development can be adjusted to lower the level of the costly yeast used in the diet formulation. In fact, previous studies have highlighted that high levels of protein in *A. ludens* artificial diets were unlikely to be fully used by the larvae [62] and that significant cost savings could be achieved at the mass-rearing facility by reducing the level of yeast in the diet formulation [25].

The protein content in excretions from third-instar larvae fed on agar and carrageenan diets increased as gel content increased, whereas, in the case of larvae fed calcium alginate diets, the protein content in excretions decreased as the gel content in the diet formulation increased (Figure 5c). The larvae of some Diptera can balance the decomposition and absorption of nutrients in their gut by regulating the secretion of digestive enzymes [63,64,65,66,67,68]. In *Anastrepha obliqua* Macquart, a closely related species to *A. ludens*, the activity of the main proteases involved in protein digestion is affected by the pH [69]. In fact, the gut of tephritid larvae is acidic [69,70], so the near-neutral pH levels in some *A. ludens* artificial diet formulations can make them unsuitable for larval development [17]. As such, another possible explanation for the unfavorable results we obtained with the calcium alginate diets we tested was that their less acidic characteristics (Figure 2d) disrupted larval nutrition, causing the low survival and low quality of flies [17].

**Figure A1:**
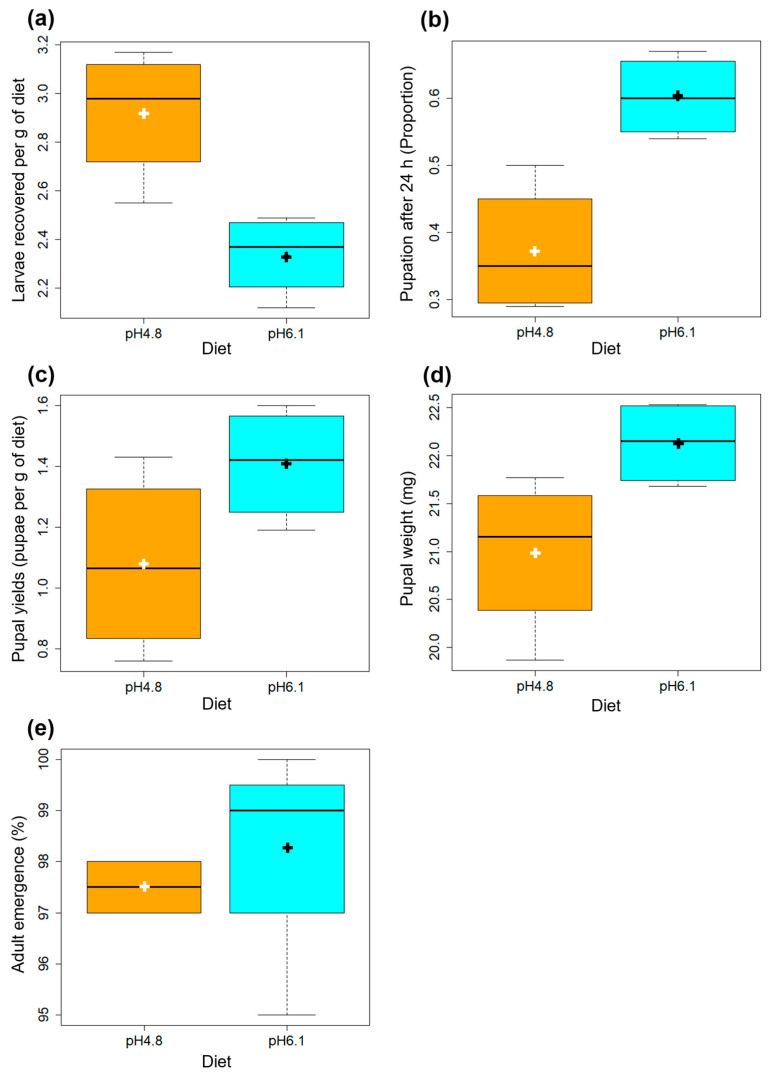
Boxplots of the production and quality parameters of *A. ludens* flies reared on calcium alginate diets with different pH values. (**a**) Larvae recovered/g of diet, (**b**) pupation after 24 h, (**c**) yield after 24 h, (**d**) pupal weight, and (**e**) adult emergence. The cross indicates mean values (n = 4).

However, the results of an additional test we performed comparing the yields and quality of flies from calcium alginate diets with pH values of 4.8 and 6.1 did not provide clear evidence that this was the case (Appendix A). On the contrary, this additional test suggested that calcium alginate diets with a mean pH value of 6.1 allowed a better development of flies to the pupal stage than diets with mean pH values of 4.8 (Figure A1b). Our results showed that *A. ludens* can be reared on calcium alginate diets with varying levels of acidity (mean pH units of 4.8, 5.3 and 6.1), but with overall less favorable results when compared with rearing on agar and carrageenan diets with a weak acidity (mean pH units of 4.2 and 4.3, respectively) (Figure 2d and Figure 3, Appendix A). Interestingly, the pH levels we found in the natural diets of *A. ludens* ranged from near-neutral pH units in the pulp of one of the preferred natural hosts (white sapote (*C. edulis*), pH 6.3) to a higher acidity level in the pulp of apple cv. ‘Golden Delicious’ (*M. × domestica*) (pH 3.6) (Figure 2d), a novel host that is starting to become infested in Mexico (M. Aluja, personal observations). These results seem to be in line with the fact that the level of acidity in the larval medium interacts in various ways with other environmental factors to affect the nutrition and development of *A. ludens* larvae [17,29].

Here, we show that the effects of larval density on the quality parameters (Figure 3f,k,l), diet removal (Figure 4b,d,e,g), and nutritional traits of flies (Figure 5b,d,f and Figure 6a–c,f) were positive, negative, or curvilinear, depending on whether the gel was agar and carrageenan or calcium alginate, which produced the firmest and most resistant to penetration diets. These findings add to those of previous studies to suggest that the response of tephritid flies to conspecific larval density conditions is rather plastic depending on the level of density and other environmental factors, such as the nutritional quality [32,33], and physicochemical characteristics of their diets [34], such as the firmness, penetration resistance, and acidity studied here. We surmise that such plasticity allows the flies to adjust their growth rates in variable environments to maximize fitness; however, this will need future confirmation.

It was noteworthy that the pulp of all the host fruit we analyzed was firmer and more resistant to penetration than the gel diets, except for calcium alginate diets whose overall mean penetration resistance was higher than that observed in the mango cv. ‘Manila’ pulp (Figure 2c). The fruit pulp with the highest firmness and penetration resistance was that of white sapote, followed by apple cv. ‘Golden Delicious’, whereas the pulp of mango cv. ‘Manila’ had the lowest firmness and penetration resistance values (Figure 2a–c). Our results on the number of larvae recovered (Figure 3a–c) being explained by the gel content and the larval density in the diet suggest that the diets that are firmer and more resistant to penetration (i.e., with higher gel content) can better support extremely high larval densities than softer diets with low levels of penetration resistance. In line with this and with the previous discussion about the cooperative feeding behavior of larvae in the face of an extremely firm and resistant to penetration medium, we note that *A. ludens* females lay more eggs in unripe mangoes with firmer pulp than in ripe mangoes with soft pulp [71]. Future examination of other physical/rheological characteristics of the host fruit of *A. ludens* (e.g., their elasticity, cohesiveness, gumminess, chewiness [72]) arising from the use of mixtures of gelling agents employed herein or other gelling/thickening hydrocolloids is warranted to better understand the factors that make fruit an optimal medium for tephritid fly larval development. This will allow the design of diets that could reproduce many of those factors. This is relevant because having a rearing system that resembles as much as possible the natural conditions required for the optimal development of the target species is key to the fitness of artificially reared insects [21]. In this regard, one advantage of the DOE and RSM experimental and modeling approach we used here is that it will allow us to calibrate the gel content in the diet formulations to reach specific firmness and penetration resistance values to mimic what *A. ludens* finds in nature in the pulp of its host fruit. It will also allow us to level down the firmness and penetration resistance of calcium alginate diets to match those values found in agar and carrageenan diets. Then, we will be able to examine what additional factors of calcium alginate diets (apart from the firmness and penetration resistance characteristics already mentioned) could limit their potential to produce large numbers of high-quality *A. ludens* individuals suitable for SIT applications.

Among such factors, we surmise that the use of CaCO_3_ in the calcium alginate diets may have affected the nutrient metabolism and functioning of flies. Dietary calcium can generate alterations in the lipid metabolism of insects [73]; here, we found that *A. ludens* larvae reared on calcium alginate diets excreted more lipids than larvae reared on agar and carrageenan diets (Figure 5a). The lower pupal weights of flies from the calcium alginate diets compared with those of flies from the agar and carrageenan diets (Figure 3g–j) might reflect a suboptimal nutritional state for the flies developed on the calcium alginate diets [11]. Larvae reared on calcium alginate diets showed a higher diet removal compared with larvae reared on agar and carrageenan diets (Figure 4c). These findings support the idea of a compensatory feeding behavior [74] in *A. ludens* larvae reared on calcium alginate diets due to lower absorption of nutrients by the insect. Interestingly, third-instar larvae from calcium alginate diets excreted lower levels of ammonium and higher levels of uric acid than larvae from agar and carrageenan diets (Figure 5e,g). Because ammonium is a by-product of protein metabolism by bacteria [75] and uric acid is a by-product of protein metabolism by larvae [76], the changes in the levels of these metabolites caused by calcium alginate in the diets suggests an alteration in protein metabolism, specifically symbiotic nitrogen fixation. Calcium ions adversely affect bacterial nitrogen fixation, as shown in model plant species [77], and symbiotic nitrogen-fixing bacteria species have been reported in tephritids such as *Bactrocera dorsalis* (Hendel), *Ceratitis capitata* (Wiedemann), and *A. ludens* [78,79,80]. However, there is still very little information on the role of the microbiota of Diptera in general and of *A. ludens* in particular in their digestion processes, nutrient absorption, development, and physical performance [81]. Dietary calcium also plays an active role in regulating the mechanical power of the indirect flight muscles in insects [82], and here we showed that flies from calcium alginate diets had a lower proportion of flying individuals compared with the flies from the agar and carrageenan diets that had no calcium (Figure 3m).

Further research is required on the cascading effects of larval density on adult nutritional physiology and life history, as our results, although not statistically clear, suggest that the larval density conditions could selectively affect adult males by decreasing their protein content (Figure 6f). More detailed studies are also required to control sources of variation not considered in the present study for the estimation of diet consumption. Here, we estimated diet removal as an indirect measurement of diet consumption. However, in our estimations we did not separate the weight of the uneaten diet from the weight of the larval excretions that could be mixed with such diet remains. Although we assume that the contribution of larval excretions to the total mass of the uneaten diet was negligible in our estimates, we recognize this is a caveat in our study that we will address in the future with a more detailed examination of excretion as a key process in the nutrition of *A. ludens* larvae. Controlling for factors such as the fecal material of the larvae in the diet and the potential effects of larvae on the rate of water loss in the diet will provide a more precise estimation of the actual diet and nutrients consumed by the larvae.

The lack of clear statistical effects for the gel type and gel content in the diets on their protein and lipid contents is not a surprising result given that all the experimental diets had the same proportions of nutritional ingredients (i.e., yeast and cane sugar) (Table 2). The positive effect of the gel content on the carbohydrate content of diets (Figure 2d) is not a surprising result either, considering that the gels we evaluated were mainly constituted by polysaccharides [38] whose monosaccharide units can be detected by the carbohydrate analysis we used [83]. A previous study showed that *A. ludens* larvae restricted to diets high in sugars relative to protein take longer to reach the pupal stage and have lower weight and reduced survival to the adult stage than flies developed on diets with balanced protein and sugar levels [32]. However, we consider that, in the present study, it is unlikely that the increase in carbohydrate content as gel content increased (Figure 2d) could have any effect on *A. ludens* larval feeding and nutrition, because the polysaccharides that compose the gelling agents we used are poorly digestible by flies, including tephritid larvae (although some gut bacteria might help to digest complex carbohydrates molecules) [84,85]. We note that although the nutritional analyses we used here are not included by the Association of Official Analytical Chemists (AOAC), they are valid, reproducible, practical, faster, require fewer samples, and are widely used methods in energetic levels research in insects [86,87].

Although in this work we found that agar and carrageenan diets outperformed calcium alginate diets for the rearing of *A. ludens*, we do not rule out the potential use of this gelling agent, since due to its characteristic of gelling at room temperature and because some SIT facilities lack the necessary infrastructure to heat large volumes of water to prepare agar or carrageenan gel diets, the use of calcium alginate could be advantageous and thus its potential use should be further investigated. An interesting quality of calcium alginate that could end up being very useful in experimentation, is the fact that one could model a natural fruit, as calcium alginate gels can be designed to harden on the exterior but remain moist/liquid in the inside. If the round ice molds used for producing round agar oviposition substrates [88] are filled with a liquid diet made with calcium alginate, one would obtain a flaccid round sphere with a hardened surface and a soft interior. If adult females could be accustomed to laying eggs into these “fruit models”, many useful experiments could be designed, as the eggs would hatch in the diet and the larvae grow as they would do in rotting pulp. However, first, a solid understanding of the system to optimize rearing in this diet will be needed. This should include reducing the costs of calcium alginate in the diet formulation, as overall, this gelling agent was more expensive than agar and carrageenan (Appendix B).

## 5. Conclusions

This study advances our understanding of gel-diet rearing systems and larval nutrition in *A. ludens*. With an advanced experimental design and statistical modeling approach, we clearly show that the agar and carrageenan diets outperformed the calcium alginate diets in basically all the production and quality parameters of flies tested. This could be in part due to (i) the firmness and penetration resistance characteristics of the calcium alginate diets limiting free movement and ad libitum feeding of larvae, (ii) the lower absorption of nutrients caused by dietary calcium, or (iii) the interference of calcium ions and alginate molecules with symbiotic nitrogen-fixing bacteria in the gut of the larvae. The adverse conditions of an extremely firm and resistant to penetration medium might be overcome by the cooperative feeding behavior of large groups of larvae and, from an applied perspective, *A. ludens* diets that are firmer and more resistant to penetration can better support extremely high larval densities than softer diets with low levels of penetration resistance. The diet consumption by larvae, nutrition, and the quality of *A. ludens* for SIT purposes are plastic parameters that vary in response to the larval density and the physicochemical characteristics of diets. This is highly relevant in a mass-rearing context, as the interaction of larval density with other dietary components can influence the number of high-quality flies that can be produced and released in SIT applications. Despite the unfavorable results we obtained with calcium alginate diets, we consider that additional research is warranted with this gelling agent, as calcium alginate exhibits some benefits (e.g., a high percentage of proteins in females and the feasibility of generating different levels of firmness in the same diet) that should be analyzed in more depth in future studies. Our study provides a useful framework for the optimization of diets for tephritid flies and other insect species.

## Figures and Tables

**Table 1:** Response variables of the experimental design with an explanation of their significance in a mass-rearing context for SIT applications.

Response Variables ^a^	Significance
Physical, chemical, and nutritional traits of the diets	
Firmness (kgF)	An indicator of diet texture that influences food intake and nutrition [15].
Penetration resistance (kgF)	An indicator of diet consistency. Influence the ability of larvae to suck the food [15].
Acidity (pH units)	pH can alter the properties of the diet, affecting feeding and causing negative effects on larval development and nutrient absorption [14,15,51].
Carbohydrates, lipids, and protein content (%)	The macronutrient content of the diet can influence the quality parameters of flies [32].
Production and quality parameters of flies	
Estimated number of larvae recovered (No.)	A successful diet must provide a greater larval yield per unit of biomass [15].
Pupation after 24 h (proportion)	Pupation is a critical step in the life history of flies, and for SIT purposes, pupae are moved to maturation after 24 h [11,26].
Pupal weight (mg)	Reflect larval nutrition and indicate pupae’s viability [11,26].
Fliers (proportion)	Flying insects are the product of mass rearing for SIT purposes. It is a critical parameter because sterile flies need to be able to fly to escape predators and find sexual partners in the field [26,29].
Diet removal and larval and adult nutritional traits	
Diet removal per larva (g)	We measured diet removed by larvae as an indirect estimation of diet consumption, which is important to determine if the diet is palatable and adequate for the development of the insect [15].
Relative consumption of carbohydrates, lipids, and proteins per larva (g)	Knowing the macronutrient content consumed per larva is essential to better understand nutrient absorption and assimilation [52].
Carbohydrates, lipids, and proteins in larvae (%)	Knowing the macronutrient content in larvae is important to know its storing and assimilation levels [53].
Carbohydrates, lipids, and proteins in larval excretions (%)	The correlation between the absorption and excretion of nutrients is associated with the quality of food [28].
Ammonia and uric acid in larval excretions (%)	Uric acid and ammonia are the nitrogen end products of protein catabolism [28].
Carbohydrates, lipids, and proteins in females and males (%)	The energy use of nutrients can be different depending on the sex by reproduction process of adult flies [54].

^a^ Details on how response variables were measured are found in Section 2.5.1. Evaluation of Response Variables.

**Table 2:** The content of gelling agents (agar, carrageenan, and calcium alginate), water, and citric acid in 15 gel diet formulations.

Diet ^a^	Gel Content in Diet (% *w*/*w*)	Gel Content in Diet (Coded Units) ^b^	Water Content in Diet (% *w*/*w*)	Citric Acid Content in Diet (% *w*/*w*) ^c^
Agar1	0.12	−1.0	79.44	0.44
Agar2	0.215	−0.5	79.34	0.44
Agar3	0.31	0.0	79.25	0.44
Agar4	0.405	0.5	79.15	0.44
Agar5	0.5	1.0	79.06	0.44
Carrageenan6	0.2	−1.0	79.36	0.44
Carrageenan7	0.3	−0.5	79.26	0.44
Carrageenan8	0.4	0.0	79.16	0.44
Carrageenan9	0.5	0.5	79.06	0.44
Carrageenan10	0.6	1.0	78.96	0.44
Calcium alginate11	0.57	−1.0	79.26	0.165
Calcium alginate12	1.0	−0.5	78.71	0.29
Calcium alginate13	1.43	0.0	78.16	0.41
Calcium alginate14	1.855	0.5	77.61	0.535
Calcium alginate15	2.285	1.0	77.05	0.66

^a^ All diets had constant levels of yeast (5.6% *w/w*) (Lake States^®^ Type BD75, Lallemand Inc S.A de C.V., USA), cane sugar (7.9% *w/w*) (Central Progreso S.A de C.V., Paso del Macho, Ver., Mexico), corn flour (6% *w/w*) (Minsa S.A. de C.V., Tlalnepantla, Edomex, Mexico), sodium benzoate (0.4% *w/w*) (Mi Granero, Alday Ingredients S.A. de C.V., SanPedro Cholula, PUE., Mexico), and methylparaben (0.1% *w/w*) (Mallinckrodt Specialty, Chemicals Co., St. Louis, MO, USA). ^b^ Coded units were used for experimental design construction and statistical analyses. ^c^ Agar and carrageenan diets had constant levels of citric acid across all gel contents tested, whereas in calcium alginate diets, the level of citric acid varied across diets as a function of the CaCO_3_ content, as explained in Section 2.3. Diets.

## Data Availability

The data presented in this study are available in Tables S1 and S3.

## Supplementary Materials

The following supporting information can be downloaded at: https://www.mdpi.com/article/10.3390/insects14120952/s1, Table S1: Data set of the response variables modelled by RSM; Table S2: Means and standard errors of each response variable for each of the gel types used in diets (without considering the effects of the gel content and larval density in the diet); Table S3: Data set of the responses of flies recorded in the additional test reported in Appendix A.

## Appendix A

This Appendix reports tests evaluating the effects of pH levels in calcium alginate diets on the production and quality parameters of artificially reared *A. ludens*.

### Appendix A.1. Preparation of Diets and Experimental Procedures

We used calcium alginate gel diets with a reduced proportion of sodium alginate:CaCO_3_ (0.91:0.09). The diets consisted of calcium alginate (1.1% *w/w*), citric acid (0.096% *w/w* and 0.44% *w/w* to achieve pH values of 6.1 and 4.8, respectively), water (78.8% *w/w* and 78.46% *w/w*), yeast (5.6% *w/w*), cane sugar (7.9% *w/w*), corn flour (6% *w/w*), sodium benzoate (0.4% *w/w*), and methylparaben (0.1% *w/w*). In the case of calcium alginate diets with pH 6.1 (i.e., 0.096% of citric acid), we first mixed all dry ingredients, except citric acid, in a domestic blender for ca. 40 s. Then, 96% of the total volume of water was incorporated into the blender and mixed with the dry ingredients for an additional 40 s. Citric acid was dissolved with the remaining 4% of the volume of water and then incorporated into the blender with the remaining ingredients and mixed for 10 s. The resulting diets were dispensed into rearing trays as described in the main text.

In the case of diets with pH 4.8 (i.e., 0.44% of citric acid), the preparation was similar but we used 92% of the total volume of water to mix all the dry ingredients, except citric acid. Then, 0.1% of citric acid was dissolved with 4% of the total volume of water and mixed with the rest of the ingredients for 40 s. The resulting diets were dispensed in rearing trays as described above and the remaining 0.34% of the citric acid previously dissolved in the remaining 4% of the total volume of water was gently poured into the diets in the trays. The diets were allowed to stand as indicated in the main text. We used portions of 150 g of diet inoculated with a fixed density of 3.33 larvae per g of diet (i.e., 500 larvae per rearing tray with 150 g of diet).

Larval bioassays were carried out as indicated in Section 2.5. Experimental Procedures of the main text. The number of larvae recovered per g of diet was estimated as the total number of larvae recovered per diet divided by 150 g of diet. Pupation after 24 h of larval separation from the diet was estimated as the quotient of the number of pupae recovered from each diet 24 h after larval separation from the diet, divided by the number of larvae recovered. Pupal yields (number of pupae/g of diet) were estimated as the number of pupae recovered per rearing tray after 24 h of larval separation from the diet, divided by 150 g of diet. Pupal weight (mg) was estimated as the average weight of samples of 100 3-day-old pupae. Adult emergence (%) was estimated based on a sample of 100 pupae. A two-sample t-test for equal variance was used to compare the means of the two treatments. Analyses were performed using R software version 4.0.3 [89].

### Appendix A.2. Results

We found higher values of larvae recovered per gram of diet in diets with pH 4.8 when compared with diets with pH 6.1 (*t* = 3.63, df = 6, *p* = 0.0109; Figure A1a). Pupation after 24 h was higher in flies from diets with pH 6.1 than in flies from diets with pH 4.8 (*t* = 3.95, df = 6, *p* = 0.0076; Figure A1b). The effects of dietary pH on pupal yields (*t* = 1.83, df = 6, *p* = 0.1162; Figure A1c), pupal weight (*t* = 2.43, df = 6, *p* = 0.0513; Figure A1d), and adult emergence (*t* = 0.65, df = 6, *p* = 0.5370; Figure A1e) were not statistically clear. The data set used for these analyses can be found in Table S3.

## Appendix B

We estimated the cost (in USD) of each gelling agent required to prepare 1 kg of each type of experimental diet (Table A1). The exchange rate at the time of writing was USD 1 = MXN 20.0925. The costs per 1 kg of agar, carrageenan, sodium alginate, and CaCO_3_ were USD 39.1, 21.3, 86.6, and 46.0, respectively. Gel costs were estimated as the proportional cost of the amount of gel required to reach specific gel contents in the diets, as indicated in Table A1. In the case of calcium alginate, its cost was the sum of the proportional costs of sodium alginate and CaCO_3_.

**Table A1:** Cost of gelling agents needed to prepare 1 kg of each experimental diet.

Diet	Gel Content (% *w*/*w*)	Gel Cost (USD)
Agar −1.0	0.12	0.05
Agar −0.5	0.215	0.08
Agar 0.0	0.31	0.12
Agar 0.5	0.405	0.16
Agar 1.0	0.5	0.20
Carrageenan −1.0	0.2	0.04
Carrageenan −0.5	0.3	0.06
Carrageenan 0.0	0.4	0.09
Carrageenan 0.5	0.5	0.11
Carrageenan 1.0	0.6	0.13
Calcium alginate −1.0	0.57	0.43
Calcium alginate −0.5	1.0	0.75
Calcium alginate 0.0	1.43	1.07
Calcium alginate 0.5	1.855	1.34
Calcium alginate 1.0	2.285	1.70
